# The Microstructure and Mechanical Properties of Si_3_N_4f_/BN/SiBCN Microcomposites Fabricated by the PIP Process

**DOI:** 10.3390/ma17102457

**Published:** 2024-05-20

**Authors:** Zhiyou Gong, Zhongkai Xu, Jian Zhang, Ruisong Guo, Yao Han, Xiaohong Sun, Zhuang Yuan, Xinqi Zhao, Bingqing Zhang, Chunming Zheng

**Affiliations:** 1 Key Laboratory of Advanced Ceramics and Machining Technology of Ministry of Education, School of Materials Science and Engineering, Tianjin University, Tianjin 300072, China; 2 Aerospace Institute of Advanced Materials & Processing Technology, Beijing 100074, China; 3 State Key Laboratory of Hollow-Fiber Membrane Materials and Membrane Processes, School of Chemical Engineering and Technology, Tiangong University, Tianjin 300387, China

**Keywords:** silicon nitride ceramic fibers, interfacial properties, polyborosilazanes, microcomposites, SiBCN

## Abstract

SiBCN ceramics based on SiC, BN and Si_3_N_4_ structures have good comprehensive properties such as high-temperature resistance, oxidation resistance, creep resistance and long life, which makes it one of the very promising ceramic material systems in military and aerospace fields, etc. In this study, SiBCN ceramics, as well as Si_3_N_4f_/BN/SiBCN microcomposites, were prepared by a polymer infiltration pyrolysis method using PBSZ as the polymer precursor. The PBSZ was completely ceramized by pyrolysis at 900 °C. The weight loss and elemental bonding forms of the products after the pyrolysis of the precursors hardly changed from 600 °C to 900 °C. After pyrolysis at 600 °C for 4 h and using the BN coating obtained from twice deposition as the interfacial phase, a more desirable weak interface of fiber/matrix with a binding strength of 21.96 ± 2.01 MPa can be obtained. Si_3_N_4f_/BN/SiBCN ceramic matrix microcomposites prepared under the same pyrolysis conditions have a relatively good tensile strength of 111.10 MPa while retaining a weak interface between the fibers and the matrix. The results of the study provide more theoretical and methodological support for the application of new composite structural ceramic material systems.

## 1. Introduction

Nowadays, faced with the rapid development of science and technology and industrial technology, the requirements for new materials in various fields are becoming more and more stringent [1,2,3,4]. Compared with traditional steel, plastic or other non-ferrous materials, ceramic materials have the advantages of high hardness, high wear resistance, high compressive strength, corrosion resistance, etc. [5]. SiBCN ceramics based on the structure of SiC, BN(C) and Si_3_N_4_ have excellent comprehensive properties such as high temperature resistance, oxidation resistance, creep resistance and a long life [6]. It can be applied in special service environments and is a very promising material system in various fields [7,8,9,10,11]. However, the inherent brittleness of SiBCN ceramic limits its application to a large extent. Ceramic matrix composites are composites of a ceramic matrix with fibers, whiskers and other reinforcements, and have a fracture toughness close to metallic materials [12,13]. Fiber-reinforced ceramic matrix composites (FRCMCs) introduce a highly efficient continuous fiber phase as a reinforcement, and the presence of fibers attenuates the crack sensitivity of the composites when subjected to applied loads, giving them excellent properties such as high strength and high toughness. They are the main candidates for components applied under extreme conditions [7,8,10,14]. The most successful toughening mechanism that occurs in FRCMCs is the deflection of matrix cracks at weak interfaces on the fiber surface. The much lower shear strength of the weak interface than that of the fiber and matrix makes cracks within the matrix more inclined to pull fibers out of the matrix as they extend to the fiber surface, which then generates a new interface at the fiber/matrix interface that deflects the cracks and absorbs the energy of crack growth, thus preventing brittle failure of the fibers and matrix [14,15,16,17,18]. However, the occurrence of the fiber pull-out phenomenon also makes the interfacial bond between the fiber and matrix need to be appropriately strong so that it can both absorb the tip stress of matrix crack propagation, which causes the fiber to pull out, and at the same time transfer the loads so that synergistic effects can be generated between the matrix and the fiber [19,20]. As a widely used weak interfacial material, BN is characterized by low hardness and low shear strength; meanwhile, its oxidation resistance has attracted much attention. In order to obtain a weak interface with appropriate interfacial bonding strength, many researchers have introduced BN coatings at the fiber/matrix interface and controlled the interfacial bonding strength by controlling the thickness of the coatings [15,16,17,21,22].

Continuous silicon nitride fiber has the advantages of high strength, excellent oxidation resistance, excellent electrical insulation and dielectric properties. It is also currently considered to be one of the most promising reinforcing fibers for composites [23,24,25]. In this study, an attempt was carried out to introduce continuous silicon nitride fibers into a SiBCN ceramic matrix. The prepared Si_3_N_4f_/SiBCN ceramic matrix composites can combine the advantages of the Si_3_N_4_ fibers and SiBCN ceramics, as well as improving the toughness of the SiBNC ceramics. However, Si_3_N_4_ fibers have a good compatibility with SiBCN ceramics, which also leads to the tendency of strong bonding at the interface between the fibers and the matrix, and cracks in the matrix tend to grow along the axial direction of the fibers, causing catastrophic damage to the composite. Therefore, in this work, boron nitride was introduced on the surface of the Si_3_N_4_ fiber as an interfacial phase using the chemical vapor infiltration (CVI) method, and then the Si_3_N_4f_/BN/SiBCN ceramic matrix composite was prepared using the PIP method [26,27,28]. Previous studies on SiBCN ceramics have focused on the effects of different preparation methods on the composites. The presence of a weak interface between the fiber and the matrix is closely related to the mechanical properties of FRCMCs. In order to achieve better mechanical properties of Si_3_N_4f_/BN/SiBCN ceramic matrix composites, in this study, we explored the effects of the precursor pyrolysis conditions and the thickness of the coating on the interfacial and mechanical properties of the composites, thus providing more theoretical and methodological support for the application of new composite structural ceramic material systems.

## 2. Materials and Methods

### 2.1. Material Preparation

In this study, the continuous silicon nitride fibers of Cansas-4101 were provided by Fujian Leadasia New Material Co., Ltd. (Ningde, China). Each bundle as-received contains 500 single fibers. The single fibers have a diameter of around 13.5 μm, a density of 2.25 ± 0.1 g cm^−3^ and a tensile strength which exceedes 1.6 GPa. Firstly, the original fibers were heat-treated at 500 °C for 30 min, thereby removing organic matter from the fiber surface. The pyrolysis experiments used a new methyl-containing liquid polyborosilazane (L-PBSZ) produced by the Institute of Chemistry, Chinese Academy of Sciences (Beijing, China), as a ceramic precursor.

### 2.2. Preparation of Composite Materials

**Figure 1:**
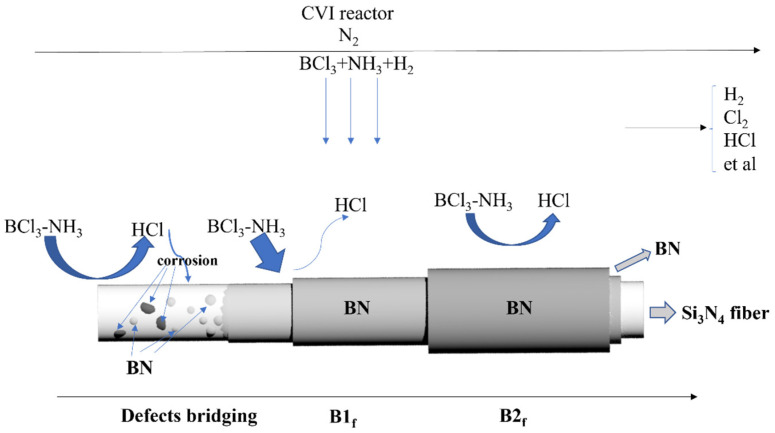
Deposition mechanism of the BN coating.

Figure 1 shows the deposition mechanism of the BN coating. In order to achieve the effect of boron nitride coating deposition and growth, the precursor gas molecules were passed into the chemical vapor reactor, reaching the vicinity of the fiber and being adsorbed on the surface of the fiber. Then, under the condition of H_2_, NH_3_ (nitrogen source), BCl_3_ (boron source) = 4:3:1 and N_2_ as dilution gas, the BN coating was obtained. BCl_3_ and NH_3_ are highly active and react rapidly and can react vigorously at room temperature. The deposition temperature was controlled at 800 °C, and the deposition time was controlled between 4 and 10 h. The specific deposition time was controlled by the weight and thickness before and after the deposition of the fiber braid body. On the fiber surface, BN coatings with various thicknesses were obtained by varying the times of the vapor deposition. For ease of reference, fibers that have undergone only one standard deposition process are named B1_f_, while fibers that have undergone *i* standard vapor deposition are named B*i*_f_.

Because of the high viscosity of the PBSZ sol, the microdrops in this study were prepared using the acupuncture needle dotting method. After straightening and fixing the stable continuous silicon nitride fiber monofilaments on an alumina crucible boat, droplets of polyborosilazane solute were adhered to the fiber surface with an acupuncture needle. The action of liquid surface tension causes the sol to form uniform droplets around the fibers. After the preparation of the microdrops was completed, the resulting samples were pyrolyzed at different temperatures and the fiber debonding tests were performed on the prepared samples.

Continuous silicon nitride fiber bundles were immersed in the PBSZ precursor sol to ensure they were moistened thoroughly between the fiber and sol. The heat-shrinkable tube was wrapped around the end of the sample and heated to shrink the sample into bundles. The sample was then solidified in a nitrogen atmosphere at 170 °C for 4 h, and pyrolyzed at different temperatures [29,30]. The obtained samples can be used to characterize performance.

### 2.3. Characterization Method

The scanning electron microscope (SEM) S4800 (Hitachi, Tokyo, Japan) was used to characterize the surface and cross-section morphology of the samples and combined with energy dispersive spectroscopy (EDS) to characterize the micro-regional elements and elemental distribution of the samples. The phase information of the samples was determined by an X-ray diffraction analyzer (Bruck, Saarbrücken, Germany, D8 Advanced). The valence information of the elements on the sample surface was determined by X-ray photoelectron spectroscopy (XPS). The weight loss curves of the PBSZ precursor sols were recorded using a Rigaku 8122 (NETZSCH, Bavaria, Germany) thermogravimetric analyzer (TGA) at a heating rate of 10 °C min^−1^ from 20 °C to 1000 °C.

### 2.4. Mechanical Property Characterization

#### 2.4.1. Mechanical Property Characterization Methods

**Figure 2:**
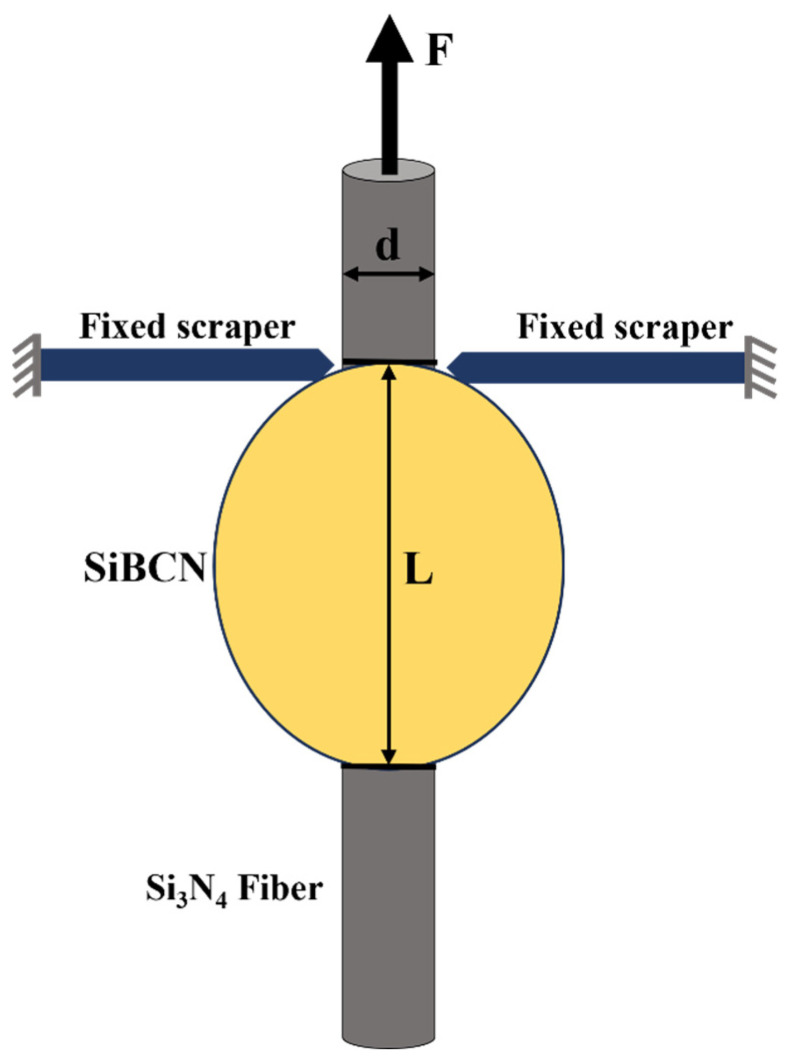
Schematic diagram of the microdrop debonding test.

The experiment of single fiber microdroplet debonding and the tensile test of the composite material at room temperature were carried out by a universal testing machine. Figure 2 shows the schematic diagram of the single fiber microdrop debonding experiment. Setting the appropriate experimental parameters, the universal testing machine applies an axial tensile stress to the sample fiber to move it upward, in the process of which a fixed scraper strips the ceramic microdroplets from the fiber surface. The required result was calculated by the load-displacement curves recorded by the sensor, and the interfacial bonding strength *τ* was calculated by Equation (1),

(1) $$\tau =\frac{F}{\pi dL},$$

The principle of the tensile test of the composite material is similar to that of the debonding test. Resin is used to fix the fiber bundles before and after pyrolysis to the surface of black cardboard. Then, the universal testing machine applies tension to both ends of the fiber composite material until the sample fails. The load displacement curves of failure of the composite fiber bundles are recorded by a sensor and the result is calculated, and the tensile strength $\sigma_{b}$ of the bundles is calculated by Equation (2),

(2) $$\sigma_{b}=\frac{F}{A}$$

where *A* is the cross-sectional area of the fiber bundle and *F* is the fracture load.

#### 2.4.2. Weibull Model

The strength and toughness of the FRCMCs depend largely on the breaking strength of the fibers. Statistical analysis through the Weibull model [31] can predict the failure behavior of the material to some extent. The two-parameter Weibull model is represented by Equation (3),

(3) $$P_{f(\sigma )}=1-\exp\left[{-\frac{L}{L_{0}}(\frac{\sigma -\sigma_{T}}{\sigma_{0}})}^{m}\right]$$

where $P_{f(\sigma )}$ is the failure probability of the fiber when the stress is less than or equal to *σ*, *L* is the scale length of the specimen, $L_{0}$ is the reference scale length (*L*/*L*_0_ = 1, a scaling constant), $\sigma_{0}$ is the characteristic failure strength, and the value of $\sigma_{T}$ is usually set to be 0 for brittle materials, and *m* is the shape parameter or Weibull modulus [32]. Taking the natural logarithm twice for both sides of Equation (3) and shifting the terms, Equation (4) can be obtained,

(4) $$\ln\ln\frac{1}{1-P_{f(\sigma )}}=m\ln\sigma -m\ln\sigma_{0}$$

The measured data are statistically analyzed. The lnln [1/(1 − $P_{f(\sigma )}$)] as the *y*-axis and the logarithmic function of the failure stress σ as the *x*-axis, and the curve formed by the two is fitted linearly. The slope of the curves obtained by fitting is the Weibull modulus *m*, and the characteristic strength $\sigma_{0}$ can be obtained from the intercept with *x*-axis. Before making the graph, we need to get the failure probability of the samples. The failure strength of *N* samples is measured experimentally, and their failure strength are ranked from smallest to largest by $\sigma_{1}$ ≤ $\sigma_{2}$ ≤ … ≤ $\sigma_{i}$ ≤ … ≤ $\sigma_{N}$.The probability of failure of the ith sample is assigned as $P_{f(\sigma_{i})}$. The function that calculates the probability of failure of the samples is the Bergman estimator (Equation (5)) [33].

(5) $$P_{f_{\left(\sigma_{i}\right)}}=\frac{i-0.5}{N+0}$$

## 3. Results

### 3.1. Effect of Coating Deposition on Fiber Strength

**Figure 3:**
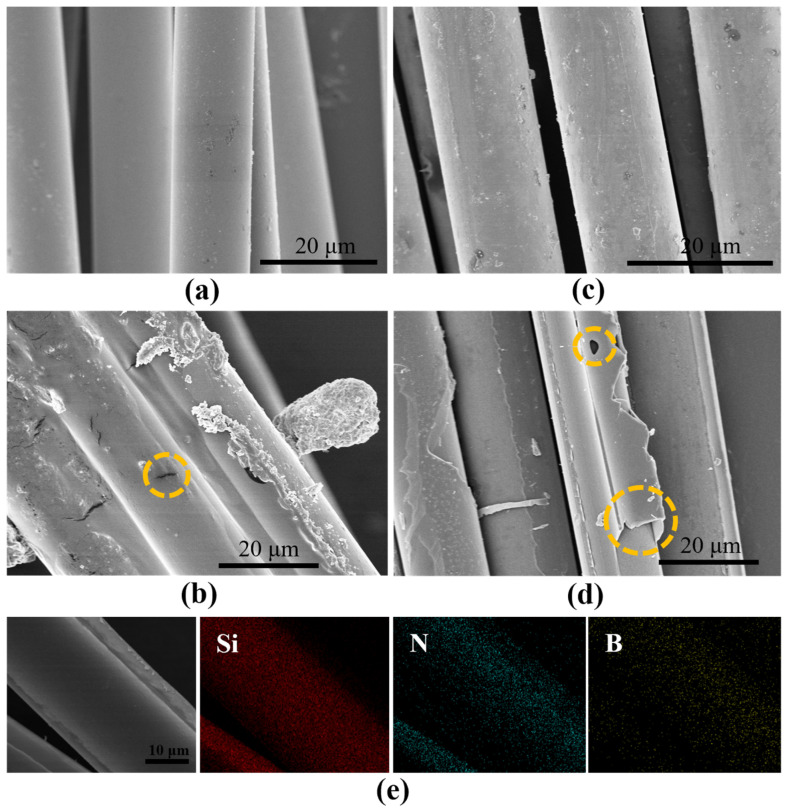
SEM and EDS images of the fiber surface before and after pretreatment: (**a**) After pretreatment and uncoated, (**b**) Before pretreatment and uncoated (cracks are shown in the orange dashed circles), (**c**) After pretreatment and coated, (**d**) Before pretreatment and coated (orange dashed circles show holes and peeling of the surface coating), (**e**) EDS images of fiber surface after pretreatment and deposition of BN.

The pretreatment of fibers is required in order to improve the deposition of the BN coating on the fiber surface. SEM images of the fibers before and after the pretreatment in a high temperature furnace are shown in Figure 3a–d. Before pretreatment, the surface of the fibers has highly noticeable uneven granular and banded residues, as seen in Figure 3b. Obvious fiber adhesion can also be observed. This results in an incomplete deposition of BN, with large holes and peeling of the coating, leaving a bare fiber surface, as shown in Figure 3d. In contrast, the coating on the fiber surface becomes more uniform, smooth and complete after pretreatment, as seen in Figure 3a,c, and is used for the subsequent preparation of FRCMCs composites. From Figure 3e, we can find that the BN was evenly coated on the fiber surface, and that the B and N elements are uniformly distributed.

In order to study the influence of coating thickness on a single fiber with different deposition thicknesses of BN, the fibers were subsequently tested for their tensile strength. From Figure S1, it can be seen that the presence of a BN coating is beneficial to the tensile strength of the fibers when the time of chemical vapor deposition is short; that is, the thickness of the coating on the surface of the fibers is thin. This is due to the fact that the process of preparing the boron nitride coating by the gas phase reaction produces a bridging effect of small molecules on the defects on the surface of the fiber. However, as the deposition time increases; that is, the thickness of the coating increases, the volumetric effect of the coating on the strength of the fibers exceeds the bridging effect of the small molecules, so the tensile strength of the fibers decreases as expected.

**Figure 4:**
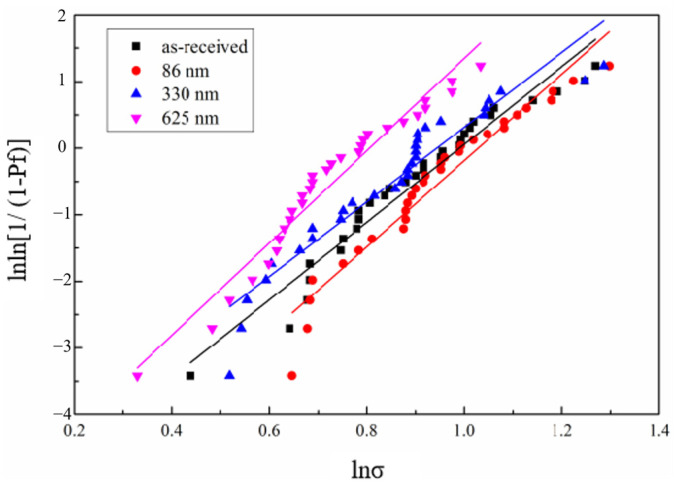
Weibull function curves of Si_3_N_4_ fibers with different BN deposition thicknesses.

**Figure 5:**
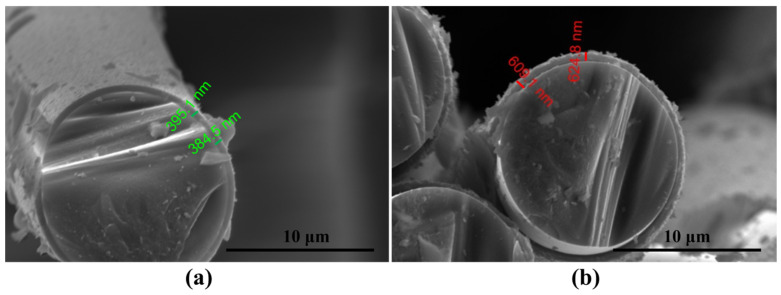
SEM images of the thickness of the deposited coating on the surface of silicon nitride fibers: (**a**) B1_f_, (**b**) B2_f_.

The experimentally obtained results of the tensile strength of the single fibers were calculated by Equation (4) and then fitted to obtain the Weibull distribution plots of the tensile strength of single fibers whose surfaces have different BN coating thicknesses (Figure 4), while their Weibull modulus can be obtained by calculation (Table 1). The average tensile strength (*σ*_mean_) and the Weibull modulus (m_*σ*_) [32] of single fibers with different BN coating thicknesses can be obtained based on the fitting results, which are calculated in Table 1. The fibers’ tensile strength dispersion is close as the coating deposition thickness is changed. Because a coating thickness of 86 nm is too thin to prevent the strong binding of the fiber matrix, the deposition condition at a BN coating thickness of 330 nm is set as the one-time standard deposition condition (B1_f_). The m_*σ*_ of B2_f_ (625 nm) was found to be the largest and the strength discreteness was the smallest. Compared to uncoated fibers, the tensile strength retention of B1_f_ and B2_f_ decreased sequentially. It is presumed that it is because the ceramic fiber has poor flexural properties, and the deposition process of the coating inevitably flexes the fibers, coupled with the influence of the bridging effect and the volume effect, which affects the distribution of the tensile strength. Figure 5 shows the enlarged SEM images of BN coatings on the surface of silicon nitride fibers with different deposition times. It can be seen that the thickness of the B1_f_ and B2_f_ surface coatings is uniform, and the morphology is complete and dense, so B1_f_ and B2_f_ are selected as the reinforcements for the composites.

### 3.2. Results of Microdroplet Debonding Experiments

#### 3.2.1. Effect of Pyrolysis on Matrix Ceramics

**Figure 6:**
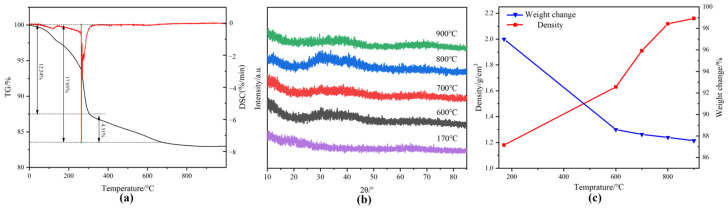
(**a**) Thermogravimetric curve of PBSZ (TG curve in black, DSC curve in red), (**b**) XRD patterns of polyborosilazane after pyrolysis at different temperatures, (**c**) Macrodensity and weight loss of PBSZ products treated with different temperatures.

The SiBCN ceramics studied in our work were obtained by PIP with PBSZ as the polymer precursor [34]. Figure 6a shows the TGA curve of the PBSZ precursor from 20 °C to 1000 °C. The weight loss of PBSZ is 12.45% from 20 °C to 350 °C, which is the main stage of the quality change of the precursor. The main reason for the weight loss at this stage is the precipitation of H_2_ and NH_3_ and other gases [35]. The weight loss of 4.51% from 350 °C to 600 °C is mainly caused by the further evolution of H_2_ and NH_3_ as well as the precipitation of C_2_H_4_ produced by the decomposition of some hydrocarbons. From 600 °C to 900 °C, the weight loss of PBSZ is 1.3%, indicating that the ceramization is basically complete and the final ceramic yield is 81.6%. Figure 6b shows XRD patterns of the precursor at different stages. Compared with the curing stage, the diffraction peaks of the product pyrolyzed at 600 °C, 700 °C, 800 °C and 900 °C for 2 h were enhanced, but no obvious crystallization peak was observed, indicating that SiBCN was amorphous. Combined with the macroscopic properties of the products (Figure 6c), it can be found that when the pyrolysis temperature is increased from 600 °C to 900 °C, the ceramic yield of the obtained products changes very little, indicating that the pyrolysis process is basically finished at 600 °C, and the main stage of the ceramization process of PBSZ is from 170 °C to 600 °C. One of the reasons for the increase in ceramic density may be the shrinkage of the material due to the increase in temperature.

**Figure 7:**
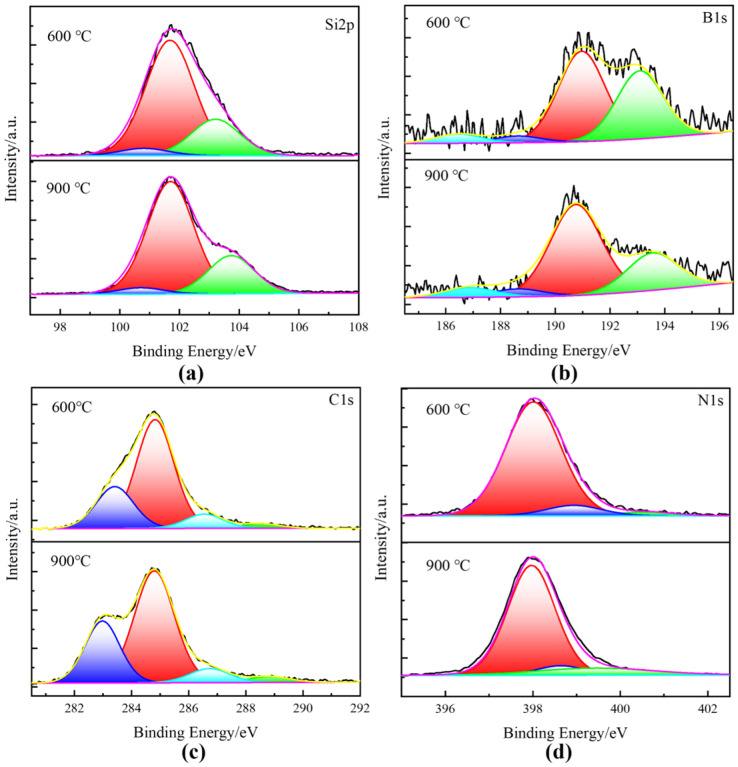
The high resolution XPS spectra of PIP-SiBCN matrix: (**a**) Si2p, (**b**) B1s, (**c**) C1s, (**d**) N1s.

Therefore, in order to gain insight into the evolution of the precursor microstructure, the elemental composition and the form of the chemical bonding of the PBSZ pyrolysis products at different temperatures were characterized by XPS. As shown in Figure S2 and Table S1, the SiBCN obtained by PIP consists of Si, B, C, N and part of O elements. The bonding states of SiBCN ceramics are shown in Figure 7a–d. The peak of elemental Si near 102 eV is fitted to the presence of three forms, Si-N (101.7 eV [29]), Si-O (103.7 eV) and Si-C (100.1 eV), where Si-N and Si-C are obtained by the breakage of the main chain of PBSZ, and the presence of the Si-O bonding is attributed to the SiO_2_ on the surface of the ceramics [36,37]. The peak of elemental B near 191 eV consists of B-N (190.9 eV), B-C (186.4 eV, 188.6 eV) and B-O (193.1 eV), and the presence of B-O is attributed to the oxidization of the sample surface [23]. The C1s spectra was fitted to obtain C-Si (283.4 eV) and C-O/N (286.6 eV). In addition, due to the presence of free carbon, graphitized carbon and adsorbed carbon, there is also C-C (284.6 eV) and O-C=O (288.5 eV) from CO_2_ [38]. The N1s spectrum can be fitted to Si-N (398.0 eV), B-N (398.4 eV) and N-O-Si (399.9 eV). It can be found that SiBCN ceramics can be obtained after pyrolysis at both 600 °C and 900 °C, and the products are mainly composed of Si-C, Si-N and B-N bonds. When the pyrolysis temperature is increased from 600 °C to 900 °C, the bonding form and content of each element do by no means change significantly. In summary, a lower temperature (600 °C) and a higher temperature (900 °C) were selected in this study for subsequent composite preparation studies.

#### 3.2.2. Characterization and Discussion of Interfacial Bond Strength at the Fiber/Matrix Interface

A micromechanical test can be used to assess the interfacial characteristics of the fiber/matrix [39,40]. The results of the fiber/matrix interfacial bond strength for the samples prepared under different conditions are shown in Table 2.

During the pyrolysis process, the uncoated fiber was in direct contact with the matrix. Strong bonding occurred between the fibers and the matrix, resulting in gas and defects generated during the ceramization process of PBSZ extending to the fiber surface, causing brittle damage at the junction, as shown in Figure S3.

After pyrolysis at the same temperature for the same amount of time, the interfacial bonding strength of the fiber/matrix increased significantly at 900 °C compared to 600 °C. As expected, the increase in the BN coating thickness also resulted in a significant decrease in the fiber/matrix interfacial bonding strength. The fiber/matrix with both once-deposited and twice-deposited BN as the interfacial coating gives a good weak interface. However, if the interfacial phase between the fiber and matrix is too thick, the synergistic effect of the two will be reduced and the reinforcing effect of the fiber will not be realized [14,41].

**Figure 8:**
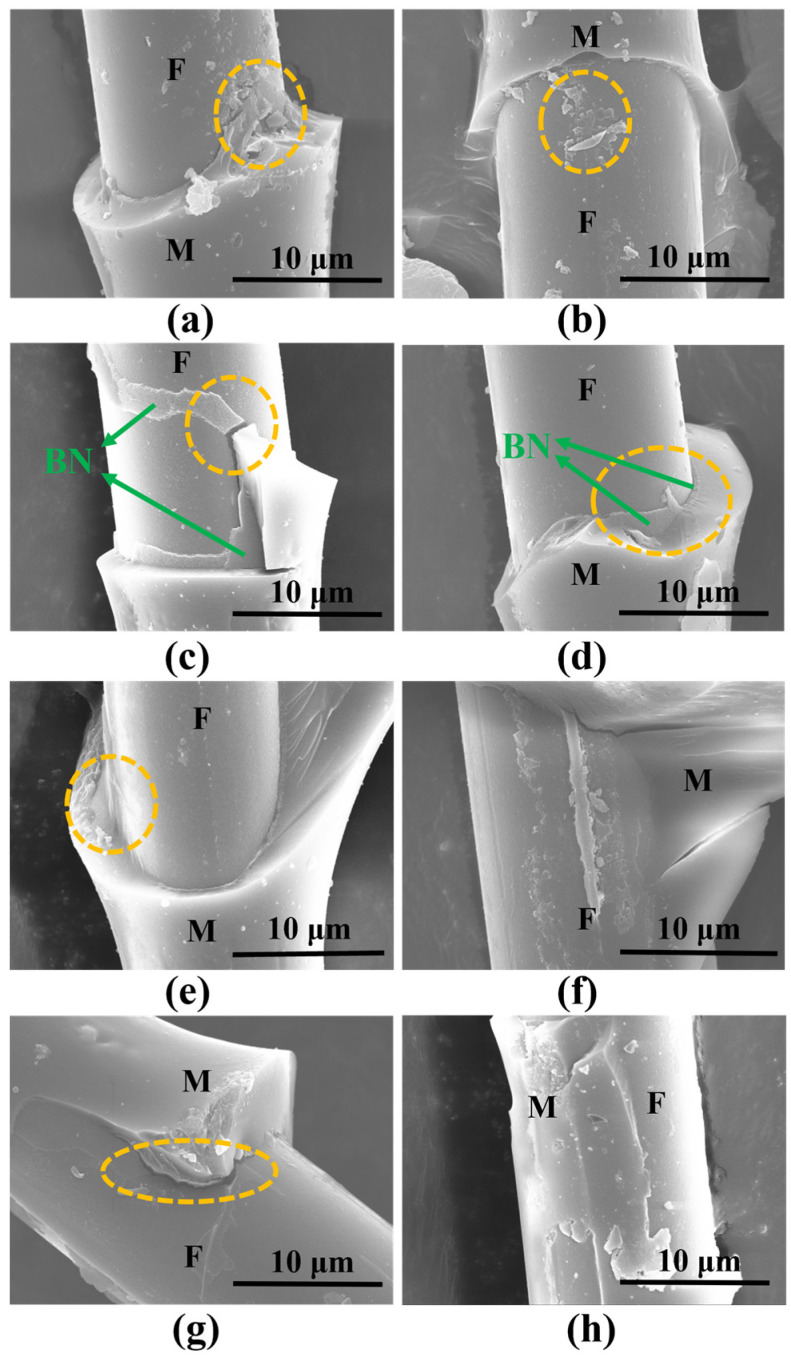
SEM images of the fiber surfaces obtained from microdroplet debonding experiments after pyrolysis of samples under different conditions (F: fiber, M: matrix, the characteristics of different F/M interfaces are shown as orange dashed circles): (**a**) 600 °C/2 h/B1_f_, (**b**) 600 °C/4 h/B1_f_, (**c**) 600 °C/2 h/B2_f_, (**d**) 600 °C/4 h/B2_f_, (**e**) 900 °C/2 h/B1_f_, (**f**) 900 °C/4 h/B1_f_, (**g**) 900 °C/2 h/B2_f_, (**h**) 900 °C/4 h/B2_f_.

The SEM images (Figure 8) of the interface of different samples were observed. After pyrolysis at 600 °C for 2 h, the surface of the B1f fiber has slight traces of erosion and damage, while the surface of the B2_f_ fiber is intact, indicating that a thick BN coating is effective in preventing direct contact and the strong bonding of fibers to the matrix after pyrolysis, which is consistent with the test results. When PBSZ was pyrolyzed at 900 °C for 4 h, there was essentially no weight loss. After the microdroplet was scraped away, as shown in Figure 8f,h, the vast and irregular fragments of the matrix were still present on the fiber surface. At this time, the fiber-matrix interface bonding strength was high, as shown in Table 2. When the pyrolysis time of the precursor was shortened to 2 h at 900 °C, the bonding strength of the fiber/matrix interface was slightly reduced. However, significant damage could still be observed on the fiber surface (Figure 8e). A microdroplet debonding test was performed on the samples after pyrolysis at 600 °C, as shown in Figure S4 for the residual fiber surface EDS map. When the coating thickness is thin, the matrix can be almost completely removed, but slight damage and residual matrix fragments can still be found on the fiber surface (Figure 8a,b). When the coating thickness increases, the smooth surface of B2_f_ and the intact coating (Figure 8c,d) indicate that BN plays a role in the interfacial phase well at this time. Combined with the interfacial bonding strength, we can find that the coating is able to prevent strong bonding between the fibers and the ceramic matrix very well. The non-loose coating structure of the fibers and the matrix also shows that it is able to transfer loads well.

### 3.3. Si_3_N_4f_/BN/SiBCN Microcomposite Strength Characterization and Discussion

We then carried out simulation and amplification experiments for the samples by preparing microcomposites to study the properties of the prepared Si_3_N_4f_/BN/SiBCN on a macroscopic scale.

The fiber bundle cross section before pyrolysis is shown in Figure S5. The fracture traces of the composite fracture show that the failure starts from a smooth semicircular plane and the cracks spread to the other side of the fracture in a divergent pattern. After crossing the midline of the section, the fracture shows a hierarchical step change, indicating the appearance of axial cracking of the fibers, which suggests that the fibers have a good stress-carrying capacity at this point and can consume more energy [42]. After pyrolysis, the composite bundle samples without a BN coating have basically no sign of fibers pulling out, and the fiber cross-section is flat and smooth, showing the characteristics of a brittle fracture.

**Figure 9:**
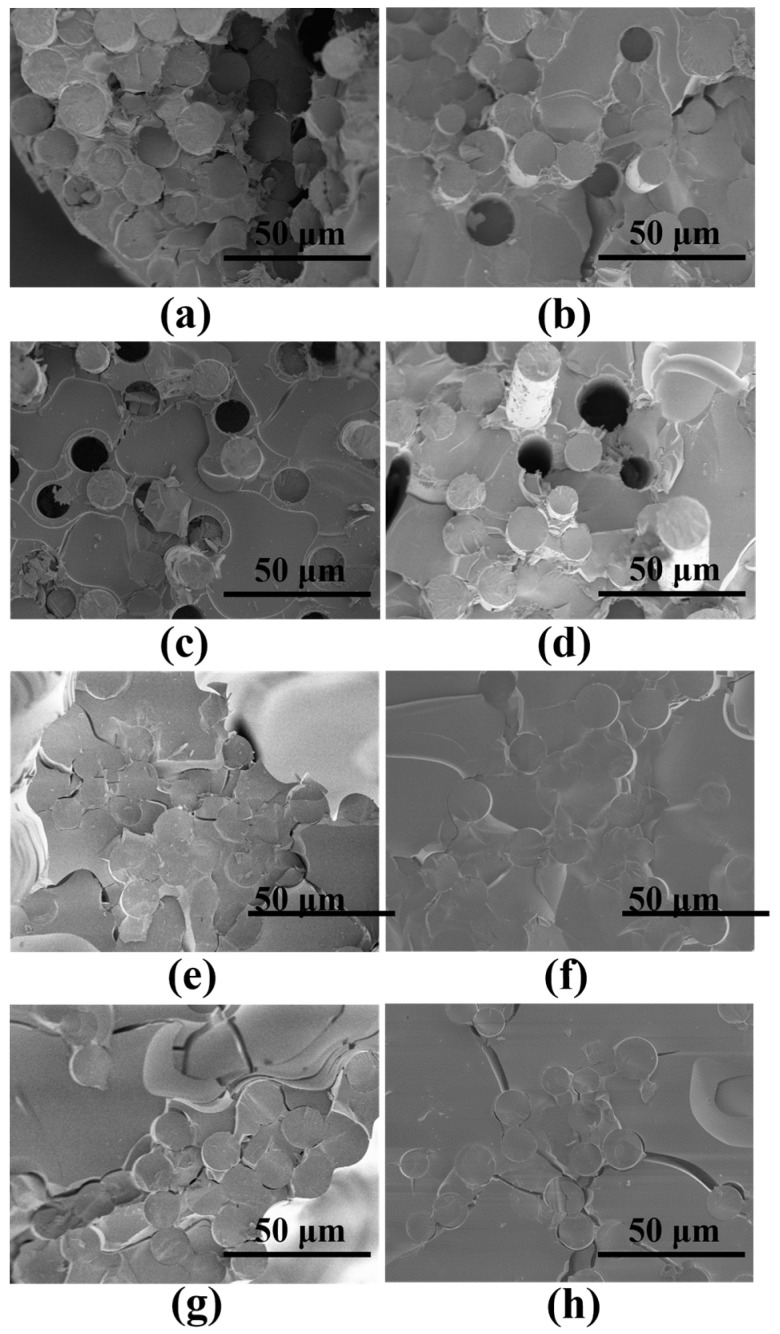
SEM images of the section morphology of the micro-composite fiber bundle: (**a**) 600 °C/2 h/B1_f_, (**b**) 600 °C/4 h/B1_f_, (**c**) 600 °C/2 h/B2_f_, (**d**) 600 °C/4 h/B2_f_, (**e**) 900 °C/2 h/B1_f_, (**f**) 900 °C/4 h/B1_f_, (**g**) 900 °C/2 h/B2_f_, (**h**) 900 °C/4 h/B2_f_.

Table 3 gives the tensile strength of composites under different pyrolysis conditions. Due to the gas release and volume shrinkage of the PBSZ precursor during the pyrolysis process, a large number of pores and cracks are generated in the SiBCN matrix (Figure S6). With the increase of the pyrolysis temperature and time, the interfacial bonding strength between the fiber and matrix increases, and the cracks are more likely to break through the BN coating to reach the surface of the fiber, as shown in the marked part of the yellow circle in Figure S6. Therefore, the tensile strength of the microcomposite obtained by pyrolysis at 900 °C is much lower than that obtained by pyrolysis at 600 °C. The section morphology of the samples with different thicknesses of BN deposition and different pyrolysis conditions are shown in Figure 9. The composite obtained by pyrolysis at 900 °C has almost no fiber pulling-out phenomenon (Figure 9e–h), which indicates that the matrix cracks spread directly to the fiber surface, causing the composite to exhibit brittle damage. In contrast, after pyrolysis at 600 °C, the composite demonstrates distinctive features of long fiber pull-out, and the residues of a coating layer can be clearly found (Figure 9a–d). The interface formed by the pull-out phenomenon can also absorb more energy and increase the strength of the composite material [43]. At a 600 °C pyrolysis condition of 2 h, the interfacial bonding strength between the fiber and matrix is too low, resulting in a poor synergistic interaction between the fiber and the matrix and a low tensile strength of Si_3_N_4f_/B2_f_/SiBCN and Si_3_N_4f/_B1_f_/SiBCN. After the coating thickness was increased, there were obvious signs of corrugated crack extension on the cross section of the extracted fibers, which was characterized by plastic fracture (Figure S7c).

When the pyrolysis time was increased to 4 h, the tensile strength of the B1_f_ and B2_f_ reinforced composites increased significantly, and there was obvious fiber pull-out phenomenon at the cross-section. The presence of matrix fragments adhering to the fiber surface also indicates that the fibers still have a certain bonding strength at this time, which can effectively carry the stress (Figure 9b,d). In this condition, compared with the B1_f_-reinforced ceramic matrix composites, the interfacial strength of Si_3_N_4f_/B2_f_/SiBCN is weaker, but the strength can be maintained at a higher level, which also indicates that the B2_f_ reinforcing body has a better reinforcing effect. According to Figure S8, the load-displacement curves of the microcomposite specimens change from linear to nonlinear with the increase of the number of BN deposition, and also show an obvious plastic damage mode, which is consistent with the experimental results.

**Figure 10:**
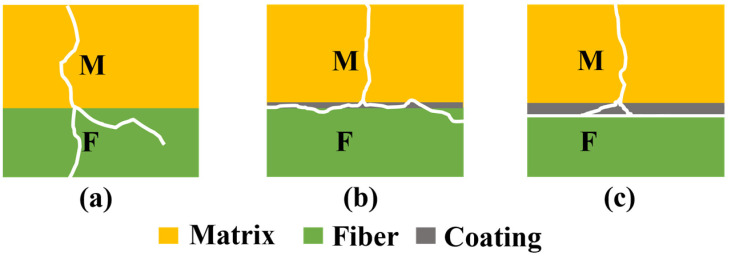
Propagation path of the cracks from fiber to matrix: (**a**) Uncoated, (**b**) B1_f_, (**c**) B2_f_.

Based on the above experimental results, we can presume the contact pattern of cracks in the matrix with fibers having different BN coating thicknesses, as shown in Figure 10 [44]. Firstly, without surface BN coating, strong bonding occurs between the fiber and matrix, and cracks reach the fiber surface directly from the matrix and pass through the fiber in the radial direction, making the composite ceramics brittle in character, as shown in Figure 10a. After one time deposition, the BN coating can effectively reduce the interfacial bonding strength between the SiBCN matrix and Si_3_N_4_ fibers. However, at this time, the BN coating is thin, and cannot completely absorb the tip stress of crack propagation and residual thermal stresses between the fibers and the matrix, and the cracks can still grow to the surface of the fibers and cause damage to the surface of the fibers, as shown in Figure 10b. The crack propagation path between the fiber and the matrix of the two-times deposition surface of BN is shown in Figure 10c. The two-times deposition can obtain a BN coating about twice the thickness of the one-time deposition, which can prevent the strong bonding between the fiber and the SiBCN matrix effectively and, at the same time, retain a degree of interfacial bonding strength. Samples with a thicker coating prevent the strong bonding between the fiber and SiBCN matrix, keeping fibers intact during the pyrolysis process. Because of the better wettability between the BN coating and the matrix, there is a higher bonding strength between the cracked BN coating and the matrix, and it is easier for the sample to pull out the fibers when subjected to load. The new interface formed can absorb the energy of the crack propagation and thermal stresses, which can lead to the deflection of the cracks.

## 4. Conclusions

In order to improve the inherent brittleness of SiBCN ceramics, in this study, SiBCN ceramic-based microcomposites toughened with silicon nitride fibers were prepared using the PIP method. The effects of precursor pyrolysis conditions and the thickness of the BN interfacial phase on the interfacial properties and tensile strength of the composites were explored by the micromechanical method. Firstly, a dense and flat BN coating with a uniform thickness was obtained by vapor deposition on the surface of the Si_3_N_4_ fibers by the CVI method at 800 °C. Compared with the single fiber tensile strength before deposition, the fiber room temperature tensile strength retention was 98.15% after one time of BN deposition on the fiber surface, and the strength retention decreased to 83.76% after two times of BN deposition. Si_3_N_4_ fibers with BN coatings deposited one time and two times on the surface were used as reinforcement, respectively. It was found that the fiber/matrix interfacial bond strength after pyrolysis increased with the increase of pyrolysis time and temperature, and the increase of the coating thickness can effectively prevent strong fiber/matrix bonding. Among them, pyrolysis at 600 °C for 4 h and using Si_3_N_4_ fibers with the BN coating deposited two times on the surface as reinforcement, a more ideal fiber/matrix weak interface can be obtained, and the interfacial bonding strength is 21.96 ± 2.01 MPa. After that, Si_3_N_4f_/BN/SiBCN ceramic matrix microcomposites were prepared. The tensile strength of the microcomposite obtained by pyrolysis at 600 °C was much higher than that of the sample obtained by pyrolysis at 900 °C. The ceramic matrix microcomposite pyrolyzed at 600 °C for 4 h with the BN coating deposited twice as the interfacial phase retained a weak interface between the fiber and the matrix, while still possessing a tensile strength of 111.10 MPa.

## Figures and Tables

**Table 1:** Fiber monofilament tensile properties.

Surface Conditions	Tensile Strength (*σ*_mean_/GPa)	Strength Retention Rate (%)	m_σ_
Uncoated	2.71 ± 0.25	—	5.9
86 nm	2.85 ± 0.19	105.2	6
330 nm	2.66 ± 0.22	98.2	5.4
625 nm	2.27 ± 0.17	83.8	6.5

**Table 2:** Interfacial bonding strength of fiber/matrix.

Pyrolysis Conditions		600 °C/2 h	600 °C/4 h	900 °C/2 h	900 °C/4 h
Samples (τ/MPa)	B1_f_	18.27 ± 5.36	26.44 ± 6.92	37.31 ± 2.64	43.65 ± 7.82
	B2_f_	17.14 ± 3.56	21.96 ± 2.00	32.46 ± 4.59	42.12 ± 7.01

**Table 3:** Tensile strength of fiber/matrix microcomposite specimens after pyrolysis.

Pyrolysis		600 °C		900 °C	
		2 h	4 h	2 h	4 h
Samples ($\sigma_{b}$/MPa)	B1_f_	74.84	115.83	11.65	4.96
	B2_f_	53.49	111.10	10.06	5.67

## Data Availability

The data presented in this study are available on request from the corresponding author due to privacy reasons.

## Supplementary Materials

The following supporting information can be downloaded at: https://www.mdpi.com/article/10.3390/ma17102457/s1, Figure S1. Strength-coating thickness distribution, from left to right: Uncoated, B1_f_, B2_f_, B3_f_, B4_f_. Figure S2. XPS spectra of the SiBCN matrix pyrolyzed at 900 °C. Table S1. Elemental compositions of PIP SiBCN determined by XPS. Figure S3. Microdroplet debonding section of uncoated fiber after pyrolysis. Figure S4. EDS images of residual interfacial element distribution for microdrop debonding of specimens without wetting agent: (a) B1_f_/600 °C/2 h, (b) B1_f_/600 °C/4 h, (c) B2_f_/600 °C/2 h, (d) B2_f_/600 °C/4 h. Figure S5. SEM images of fiber bundle filament sections: (a) B1_f_-sample before pyrolysis, (b) B2_f_-sample before pyrolysis, (c) Si_3_N_4f_-sample after pyrolysis, (d) Single fiber section of B1_f_-sample, (e) Single fiber section of B2_f_-sample, (f) Single fiber sections of Si_3_N_4f_-sample. Figure S6. SEM images of crackes formation and propagation of composite materials: (a) Section of microcomposite sample, (b) Crack propagation. Figure S7. SEM images of single fiber pull-out section morphology of microcomposite sample. Figure S8. Tensile load-displacement curves of microcomposite sample.
