# Supplementary material for: The Microstructure and Mechanical Properties of Si3N4f/BN/SiBCN Microcomposites Fabricated by the PIP Process
Source: Materials (Basel). 2024 May 20;17(10):2457. doi: 10.3390/ma17102457 (PMC11122856; doi:10.3390/ma17102457)
Supplement: Supplementary file 1 [file materials-17-02457-s001.zip › materials-2973368-supplementary.pdf]

Supporting Information

Microstructure and Mechanical Properties of Si_3N_{4f}/BN/SiBCN Microcomposites Fabricated by Polymer Infiltration and Pyrolysis

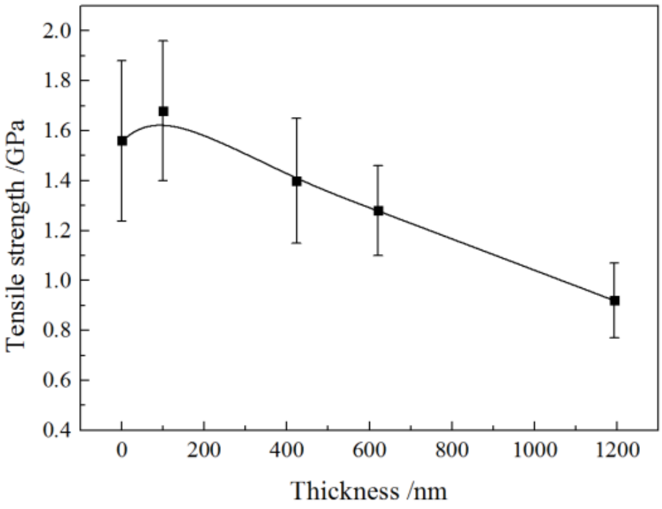

Figure S1. Strength-coating thickness distribution, from left to right: Uncoated, $B1_f$, $B2_f$, $B3_f$, $B4_f$.

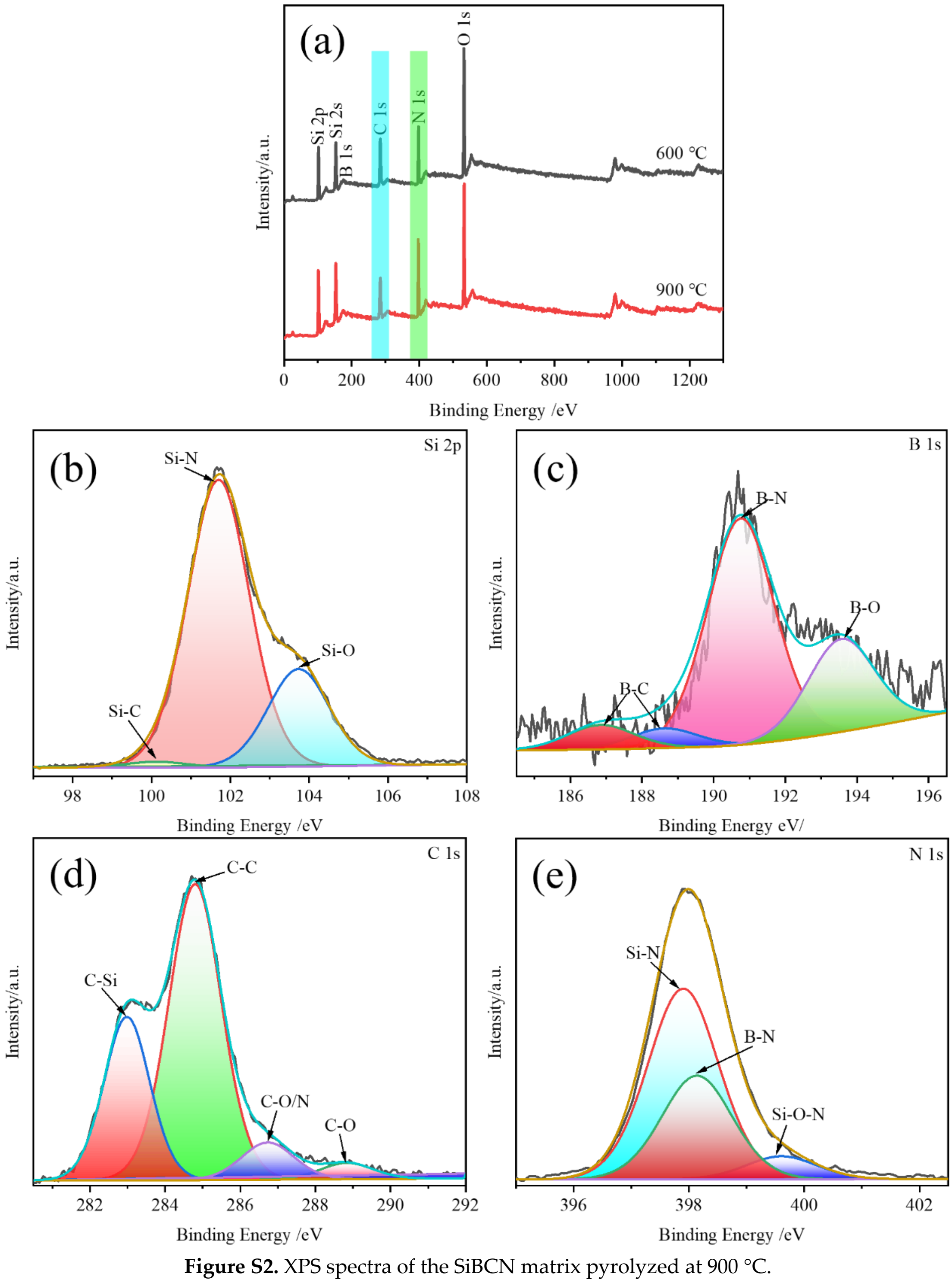

Figure S2. XPS spectra of the SiBCN matrix pyrolyzed at 900 °C.

Table S1. Elemental compositions of PIP SiBCN determined by XPS.

Sample	at%				
	Si	B	C	N	O
PIP SiBCN	37.77	4.38	26.34	16.74	14.76

With C1s as the reference spectrum, the carbon element on the fiber surface is primarily free carbon (284.6 eV, Figure S2d). The Si element on the fiber surface exists in three states

(Figure S2b). The Si-N bond in silicon nitride itself has an energy of 101.7 eV. What’s more, Si is attributed to both nitrogen and oxygen at 102.3 eV and to Si-O bonds at 103.5 eV, showing that the fiber oxidizes in an atmosphere with air. The N element exists as an N-Si bond (397.4 eV, Figure S2d), which corresponds to the previous Si2p fitting peak.

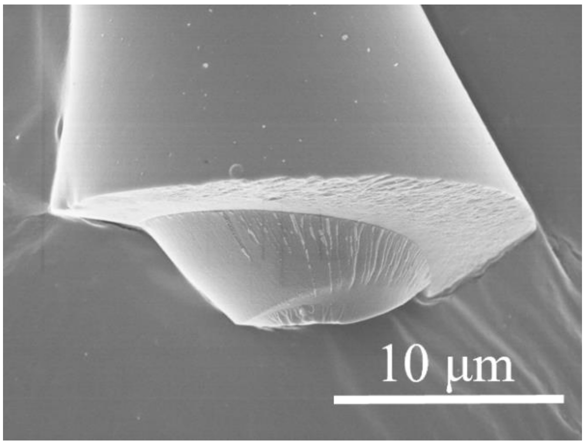

Figure S3. Microdroplet debonding section of uncoated fiber after pyrolysis.

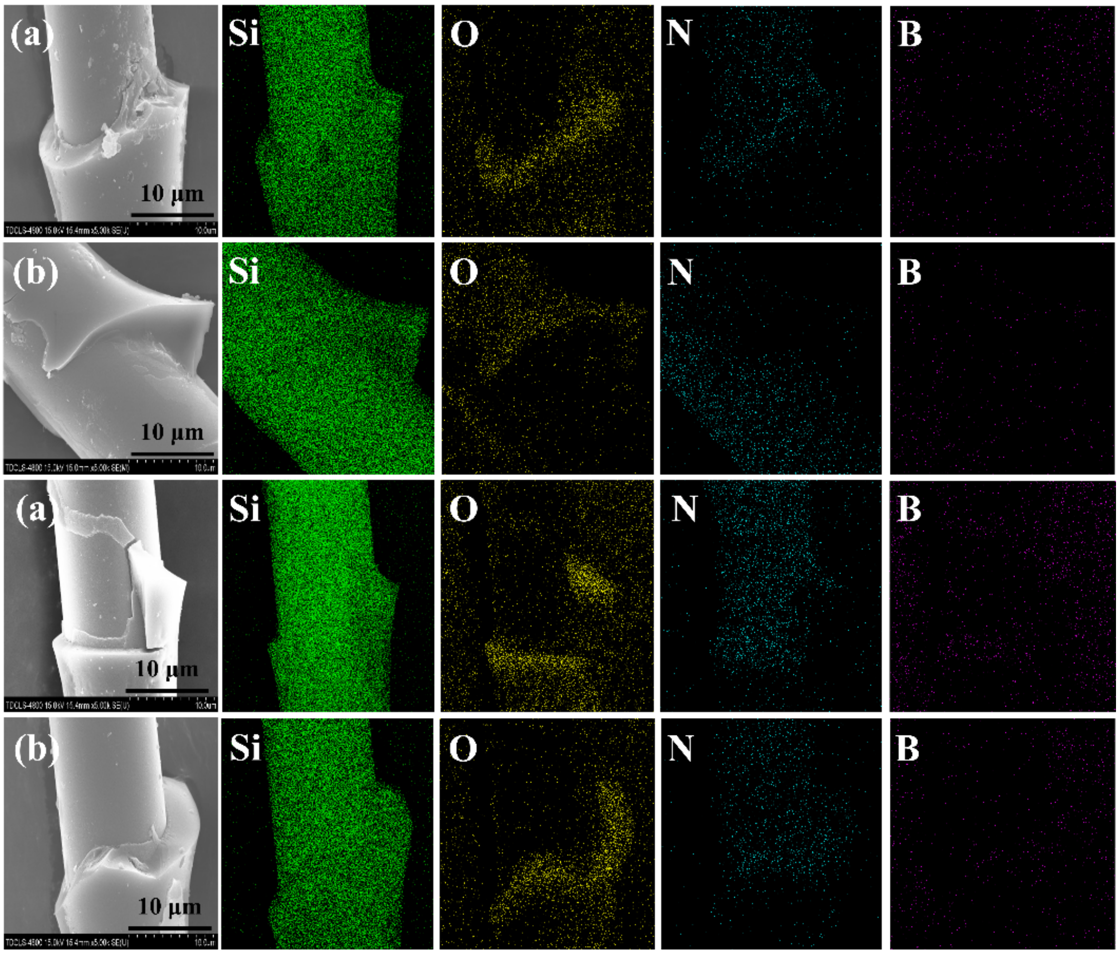

Figure S4. EDS images of residual interfacial element distribution for microdrop debonding of specimens without wetting agent: (a) $B1_f$ /600 °C/2 h, (b) $B1_f$ /600 °C/4 h, (c) $B2_f$ /600 °C/2 h, (d) $B2_f$ /600 °C/4 h.

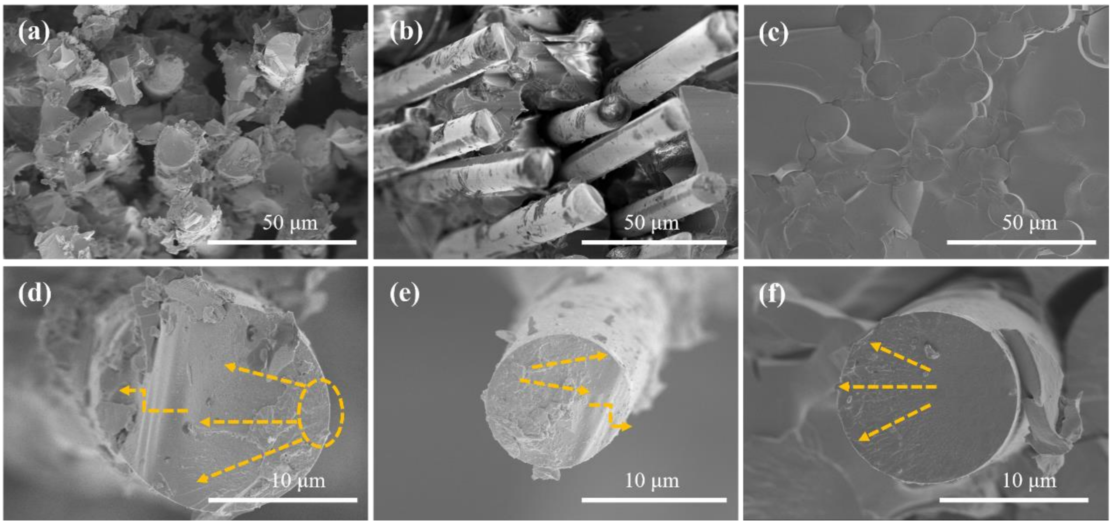

Figure S5. SEM images of fiber bundle filament sections: (a) $B1_f$-sample before pyrolysis, (b) $B2_f$-sample before pyrolysis, (c) Si_3N_{4f}-sample after pyrolysis, (d) Single fiber section of $B1_f$-sample, (e) Single fiber section of $B2_f$-sample, (f) Single fiber sections of Si_3N_{4f}-sample.

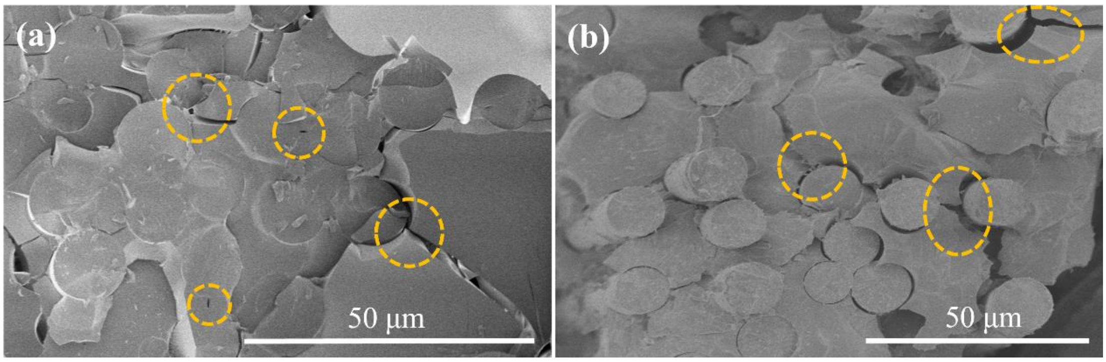

Figure S6. SEM images of crack formation and propagation of composite materials: (a) Section of the microcomposite sample, (b) Crack propagation.

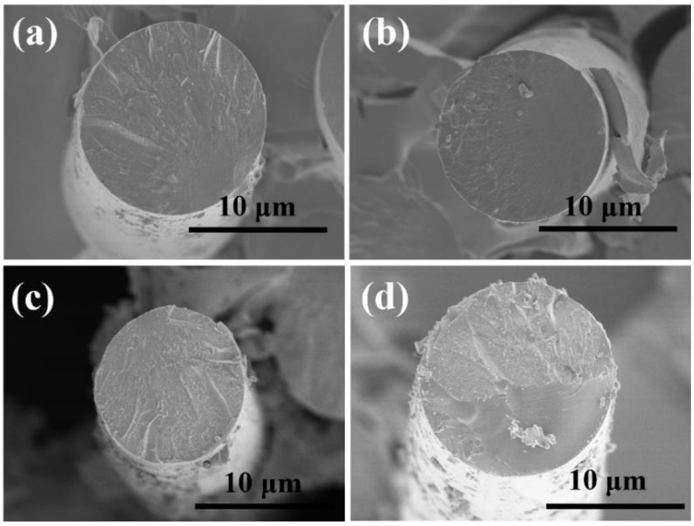

Figure S7. SEM images of single fiber pull-out section morphology of the microcomposite sample.

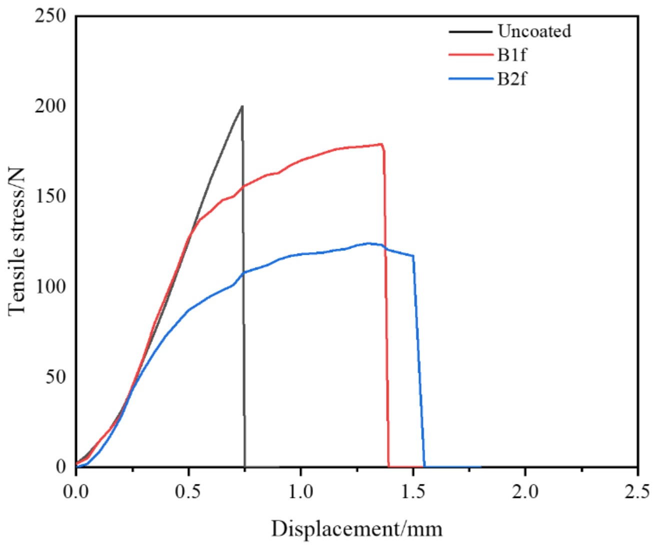

Figure S8. Tensile load-displacement curves of the microcomposite sample.
